# Tremor in Vascular Cognitive Impairment Raises the Possibility of Mixed Pathology With Lewy Body Disease

**DOI:** 10.3389/fnins.2020.00781

**Published:** 2020-07-29

**Authors:** Pai-Yi Chiu, Ray-Chang Tzeng, Cheng-Yu Wei, Guang-Uei Hung, Chaur-Jong Hu

**Affiliations:** ^1^Department of Neurology, Show Chwan Memorial Hospital, Changhua, Taiwan; ^2^Department of Nursing, College of Nursing and Health Sciences, Da-Yeh University, Changhua, Taiwan; ^3^Department of Neurology, Tainan Municipal Hospital (Managed by Show Chwan Medical Care Corporation), Tainan, Taiwan; ^4^Department of Exercise and Health Promotion, College of Education, Chinese Culture University, Taipei, Taiwan; ^5^Department of Nuclear Medicine, Chang Bing Show Chwan Memorial Hospital, Changhua, Taiwan; ^6^Department of Neurology, Taipei Medical University-Shuang Ho Hospital, School of Medicine, College of Medicine, Taipei Medical University, New Taipei City, Taiwan

**Keywords:** tremor, vascular cognitive impairment, Lewy body disease, risk factors, neuroimaging

## Abstract

**Objectives:**

Tremor is common in patients with Lewy body disease (LBD) and not rare in normal individuals. Prevalence of tremor in patients with vascular cognitive impairment (VCI) and its association with other comorbidities are seldom studied. The aim of this study was to investigate the patient characteristics of VCI associated with tremor and to evaluate the possibility of mixed pathology with LBD in these patients.

**Methods:**

Retrospective analysis of a large population with VCI registered in the database of a regional healthcare system was performed. VCI patients were divided into tremor and non-tremor groups. The associated characteristics including demographics, clinical features in motor and non-motor domains, vascular risk factors, and neuroimaging features were compared between the tremor group and the non-tremor group.

**Results:**

Among 1337 patients with VCI, 292 (21.8%) had tremor, while 1045 (78.2%) did not have tremor. The tremor group had significantly higher prevalence of all motor and non-motor LBD clinical features than the non-tremor group. The tremor group also demonstrated more severe neuropsychiatric symptoms. Among patients with tremor, patients having tremor onset earlier than stroke onset showed significantly higher prevalence of rapid eye movement sleep behavior disorder. All comparisons were adjusted for age and severity of dementia.

**Conclusion:**

Tremor is a common comorbidity of VCI. VCI patients with tremor had a higher prevalence of motor and non-motor LBD features. These findings raised the possibility of VCI patients with tremor having high possibility of mixed pathology with LBD.

## Introduction

Tremor is a common symptom/sign in patients with neurological as well as systemic disorders, and it occurs primarily during action or at rest (Bhatia et al., 2018). Tremor at rest is among one of the most important clinical features of Lewy body disease (LBD) including Parkinson’s disease (PD) and dementia with Lewy bodies (DLB) (Postuma et al., 2015; McKeith et al., 2017). However, prevalence of resting or kinetic tremors in patients with vascular cognitive impairment (VCI) and their association with the comorbidities are seldom studied. A previous study showed that movement disorders were uncommon in stroke patients and only 1% of the stroke patients developed movement disorders after acute stroke (Ghika-Schmid et al., 1997). Atypical tremors are observed more commonly after acute stroke than typical action or resting tremors (Ghika-Schmid et al., 1997; Handley et al., 2009). Locations of brain lesions in tremor resulting from acute stroke were varied and included the cortical, subcortical, and cerebellar regions (Handley et al., 2009). Stroke in the midbrain, basal ganglia, or chronic small vessel cerebrovascular disease can result in acute onset of PD-like movement disorders or parkinsonism. This condition is termed “vascular parkinsonism” (van Zagten et al., 1998; Handley et al., 2009). A study of vascular parkinsonism revealed that one or more parkinsonian signs were observed in 36% of the patients with small vessel cerebrovascular disease, and clinically, 11% of those patients had parkinsonism (van Zagten et al., 1998). Despite the clinical presentation and association of VCI with vascular parkinsonism that has been documented, very few studies have focused on the comorbidity of tremor in VCI. A previous study on parkinsonian features including tremor among patients with mild cognitive impairment (MCI), vascular MCI, and subcortical vascular dementia revealed that prevalence of tremor was not different among these groups (Frisoni et al., 2002). Tremors as a comorbidity of stroke with or without cognitive impairment are not typical features of vascular parkinsonism. The predominant clinical feature in most cases of vascular parkinsonism is gait impairment rather than tremor (Korczyn, 2015). Therefore, according to the current evidence, the underlying mechanism of tremor associated with VCI is still unclear and deserves further investigation and clarification.

Mixed dementia have traditionally focused on the coexistence of Alzheimer’s disease (AD) and CVD in the same demented patient (Langa et al., 2004; Custodio et al., 2017). However, pathological study did show that in persons with dementia, over 50% had multiple diagnoses including AD, PD/LBD, or CVD (Schneider et al., 2007). Validation studies on DLB consensus criteria have provided evidence with high specificity based on the characteristic clinical Lewy body features by comparing the pathological findings (Nelson et al., 2010; Skogseth et al., 2017). A study on AD with typical AD pathology also showed that Lewy-type synucleinopathy influences motor and non-motor clinical findings in AD patients (Savica et al., 2019). Multiple pathological processes are common in dementia patients, and they can affect the clinical presentations (Woodward et al., 2010). Based on these findings showing a high association of clinical Lewy body features with Lewy body pathologies in LBD as well as in non-LBD patients, we proposed that the presentation of tremor should raise the possibility of co-existing LBD pathophysiology in patients with VCI (Postuma et al., 2015; McKeith et al., 2017; Bhatia et al., 2018). Therefore, the present study aimed to investigate the prevalence of tremor and the characteristics of VCI patients in various cognitive states for exploring the possibility of co-existing pathophysiology of LBD by observing and comparing the prevalence of clinical Lewy body features among patients with and without tremor.

## Materials and Methods

### Database

This retrospective study selected participants registered in a dementia database of a healthcare system in Taiwan. From 2015/10/1 to 2019/9/30, a total of 6353 participants were registered in the database, and those who met the diagnosis of VCI (*n* = 1337) were selected and analyzed. Normal elderly (*n* = 573), patients with non-vascular MCI (*n* = 766), AD (*n* = 1867), DLB (*n* = 456), PD (*n* = 334), PD dementia (*n* = 309), essential tremor (*n* = 311), normal pressure hydrocephalus (*n* = 180), frontotemporal dementia (*n* = 84), other or undetermined dementia (*n* = 1475) were excluded from the study. Only the patients who had undergone at least a cerebral computed tomography scan or cerebral magnetic resonance imaging (MRI) and a set of blood screening tests for ruling out other possibilities of cognitive decline that could be analyzed were included in the study. The following information recorded in the database was used in this study:

1. Age, gender, education, duration of motor dysfunction and cerebrovascular disease (CVD), subtypes of CVD, and current medication at the time of entry.
2. Dementia severity according to Clinical Dementia Rating Scale (CDR) (Morris, 1993).
3. A structured questionnaire including motor features (resting tremor, postural/action tremor, bradykinesia, rigidity, postural instability, asymmetric onset, and easily falling tendency) and non-motor features [cognitive fluctuation, parkinsonism, visual hallucinations (VHs), rapid eye movement (REM) sleep behavior disorder (RBD), severe neuroleptic sensitivity (SNS), depression, apathy, delusions, urinary frequency, incontinence, and constipation] for the diagnosis of DLB (Chiu et al., 2019).
4. Assessment of activities of daily living according to the Instrumental Activities of Daily Living (IADL) scale (Lawton and Brody, 1969).
5. Cognitive performance according to the Cognitive Abilities Screening Instrument, Chinese version (CASI C-2.0) (Lin et al., 2002) and the Montreal Cognitive Assessment (MoCA) (Nasreddine et al., 2005).
6. Neuropsychiatric symptoms according to the 12-item version of the Neuropsychiatric Inventory (NPI) on the basis of observations within the past month (Cummings, 1988).
7. Clinically relevant vascular risk factors (VRFs) including hypertension, arrhythmia, coronary artery disease, diabetes, hyperlipidemia, and cerebrovascular disease (history of stroke/transient ischemic attack or the diagnosis of vascular encephalopathy in brain imaging).
8. A set of blood screening tests including complete blood count, COT, GPT, BUN, creatinine, total cholesterol, TG, LDL, ac glucose, HbA1c, TSH, free T4, RPR, vitamin B12, and folic acid.

### Assessment of Disease Severity and Subtypes

VCI in this database was diagnosed according to the criteria for probable vascular dementia, possible vascular dementia, and vascular MCI in the 2011 American Heart Association/American Stroke Association criteria for VCI (Gorelick et al., 2011). A diagnosis of DLB and its core clinical features was made according to the revised consensus criteria for probable DLB developed by the fourth report of the DLB consortium (McKeith et al., 2017). Fluctuation was diagnosed when a Mayo Fluctuation Composite Score (MFCS) > 2 (Ferman et al., 2004) was present. VHs were diagnosed when a clinical history of recurrent complex VHs were present. RBD was diagnosed when the minimal criteria for REM sleep behavior disorder according to the International Classification of Sleep Disorders, revised (American Academy of Sleep Medicine, 2001) was met. SNS was diagnosed when a clinical history was established for both the usage of neuroleptic drugs and an obvious association of adverse events with the neuroleptic drugs. Depression, apathy, and delusions were diagnosed by NPI. Urinary frequency, incontinence, and constipation were diagnosed according to chart recording. Cognitive tests of all patients were performed by a trained neuropsychologist. Severity and subtypes of dementia were diagnosed by a consensus meeting composed of neurologists, neuroradiologists, nuclear medicine doctors, and neuropsychologists.

### Data Analysis

The Chinese version of SPSS 22.0 for Windows (IBM SPSS Statistics, IBM Corp., Armonk, NY, United States) was used for statistical analyses. Comparisons of the demographic data including CDR, clinical features, IADL, CASI, MoCA, and composite scores (frequency vs. severity) of the NPI were performed between the tremor and the non-tremor VCI groups using the independent *t*-test and odds ratios (ORs) were adjusted for age and dementia severity according to CDR. VCI subtypes and CVD subtypes were analyzed using the chi-squared test. Clinical history of VRFs, current use of antipsychotics, and current use of antiparkinsonian drugs were analyzed using the chi-squared test and all ORs were adjusted for age and dementia severity according to CDR. To compare the associations of clinical motor and non-motor features and neuroimaging variables between the tremor group and the non-tremor group, all ORs were adjusted for age and dementia severity according to CDR.

### Ethical Considerations

The participants were selected from a dementia database of Show Chwan Healthcare System. The study design was retrospective and the data were analyzed anonymously. The Committee for Medical Research Ethics of Show Chwan Memorial Hospital reviewed the project and the Data Inspectorate approved the study.

## Results

Among 1337 patients with VCI, 292 (21.8%) had tremor, while 1045 (78.2%) did not have tremor. In the tremor group, 48 (16.4%) had isolated resting tremor, 99 (33.9%) had isolated postural/action tremor, and 145 (49.7%) had resting as well as postural/action tremors. The tremor group had lower educational levels (4.1 vs. 5.0 years, OR = 0.95, *p* = 0.004), longer duration of motor dysfunction (4.2 vs. 2.6 years, OR = 0.95, *p* < 0.001), and more severe neuropsychiatric symptoms (NPI scores: 11.6 vs. 8.3, OR = 1.03, *p* < 0.001) than the non-tremor group. Gender, duration of CVD, IADL, CASI, MoCA, VCI subtypes, and CVD subtypes showed no significant difference between the tremor group and the non-tremor group. Cognitive domains of CASI as well as MoCA were compared between tremor and non-tremor groups and showed no significant difference. Comparisons of the demographic data are summarized in Table 1.

**TABLE 1:** Background characteristics of tremor group compared to non-tremor group among VCI patients adjusted for age and dementia severity by CDR.

	Tremor *Mean* (*SD*, range)	Non-tremor *Mean* (*SD*, range)	OR/^*^χ^2^	*P*
*n*	292	1045		
Age, year	76.2 (9.1, 28–94)	75.7 (7.9, 40–102)	NA	
CDR 0.5/1/2/3, *n*	78/84/81/49	364/282/240/159	NA	
Gender, f/m, *n*	149/143	514/531	1.03	NS
Education, year	4.1 (4.2, 0–16)	5.0 (4.6, 0–18)	0.95	0.004
**Disease duration, year**				
CVD	3.2 (4.6, 0.1–20.0)	3.3 (5.6, 0–60)	1.00	NS
Motor dysfunction	4.2 (6.6, 0–60)	2.6 (3.7, 0–30)	1.07	<0.001
IADL	2.3 (2.9, 0–8)	2.8 (3.1, 0–8)	0.96	NS
MoCA	7.6 (6.7, 0–27)	8.2 (7.3, 0–29)	1.00	NS
CASI	42.3 (25.9, 0–94)	43.1 (25.5, 0–94)	1.01	NS
NPI	11.6 (12.1, 0–87)	8.3 (10.0, 0–87)	1.03	<0.001
VaMCI/PrVaD/PoVaD	31/139/122	152/521/372	5.17*	NS
CVD Subtypes	Mi 30/St 41/Sc 61/	Mi 121/St 173/Sc 255/	11.92*	NS
	Bw 77/Cc 46/He 16/	Bw 195/Cc 197/He 46/		
	Ot 21	Ot 58		

VCI, vascular cognitive impairment; CDR, Clinical Dementia Rating scale; n, number of cases; NA, not applicable; NS, non-significance; OR, odds ratio; CVD, cerebrovascular disease. Motor dysfunction includes any of the following symptoms: tremor, rigidity, bradykinesia, postural instability, hypotonic speech, or easily falling. IADL, Instrumental Activities of Daily Living; MoCA, Montreal Cognitive Assessment; CASI, Cognitive Abilities Screening Instrument; NPI, total score of 12-domain Neuropsychiatric Inventory; VaMCI/PrVaD/PoVaD, vascular mild cognitive impairment/probable vascular dementia/possible vascular dementia. Mi, multi-infarct; St, strategic infarct; Sc, subcortical lacunes; Bw, Binswanger disease; Cc, complex combination; He, hemorrhage; Ot, others. *Chi-square value without adjustments.

The comparison of Lewy body clinical motor features revealed that the tremor group had significantly higher prevalence of all motor features (*p* < 0.001 for bradykinesia, rigidity, postural instability, hypotonic/monotonic speech, and easily falling tendency) than the non-tremor group after adjustment for age and disease severity according to CDR. The comparison of Lewy body non-motor features showed that the tremor group had significantly higher prevalence of all non-motor features (*p* < 0.001 for fluctuation of cognition, VHs, RBD, SNS, depression, urinary frequency, constipation, and incontinence) than the non-tremor group after adjustment for age and disease severity according to CDR. Patients in the tremor group also demonstrated significantly higher prevalence of delusions (*p* = 0.001) and apathy (*p* = 0.015). Clinically, At least two DLB core features and/or indicative biomarkers are essential for the diagnosis of probable DLB. Our analysis and comparison between the two groups showed that the tremor group had significantly higher probable DLB standard compliance than the non-tremor group (OR = 5.36, *p* < 0.001) (Table 2).

**TABLE 2:** Motor and non-motor features of tremor group (*n* = 292) compared to non-tremor group (*n* = 1045) adjusted for age and dementia severity by CDR.

	Tremor, *n* (%)	Non-tremor, *n* (%)	OR	*p*
**Motor features**				
Bradykinesia	182 (62.3)	283 (27.1)	4.44	<0.001
Rigidity	156 (53.4)	214 (20.5)	4.39	<0.001
Postural instability	213 (72.9)	431 (41.2)	3.80	<0.001
Hypotonic speech	90 (30.8)	158 (15.1)	2.46	<0.001
Easily falling	59 (20.2)	103 (9.9)	2.26	<0.001
Parkinsonism*	236 (80.8)	234 (22.4)	14.51	<0.001
**Non-motor features**				
Fluctuation	126 (43.2)	287 (27.5)	2.01	<0.001
VHs	56 (19.2)	101 (9.7)	2.12	<0.001
RBD	42 (14.4)	72 (6.9)	2.19	<0.001
SNS*	9 (45.0)	6 (20.0)	4.25	<0.001
Delusions	65 (22.3)	139 (13.3)	1.80	0.001
Depression	80 (27.4)	178 (17.0)	1.88	<0.001
Apathy	50 (17.1)	117 (11.2)	1.57	0.015
Urinary frequency	58 (20.0)	93 (9.0)	2.62	<0.001
Constipation	67 (23.1)	132 (12.8)	2.10	<0.001
Incontinence	65 (22.3)	133 (12.7)	1.92	<0.001
DLB core features + indicative biomarkers ≧ 2	155 (53.1)	189 (18.1)	5.36	<0.001

n, number of cases; CDR, Clinical Dementia Rating scale; OR, odds ratio; VHs, visual hallucinations; RBD, REM sleep behavior disorder; SNS*, Severe neuroleptics sensitivity among 50 patients ever receiving antipsychotics; Parkinsonism*, at least two parkinsonian motor symptom/sign.

Table 3 demonstrates the comparisons of the relationship of CVD and dementia, VRFs, and current medication between the tremor group and the non-tremor group. After adjustment for age and dementia severity according to CDR, the tremor group had significantly higher prevalence of all VRFs including hypertension, diabetes, hyperlipidemia, and heart disease (all *p* values ≤ 0.001). Comparison of current medications revealed that the tremor group showed higher consumption of antiparkinsonian drugs (*p* < 0.001) and antipsychotics (*p* = 0.002) than the non-tremor group.

**TABLE 3:** Relationship of CVD and dementia, vascular risk factors, and current medication of tremor group (*n* = 292) compared to non-tremor group (*n* = 1045) adjusted for age and dementia severity by CDR.

	Tremor, *n* (%)	Non-tremor, *n* (%)	OR	*p*
**Relationship of CVD and dementia**				
History of CVD	210 (71.9)	780 (74.6)	0.82	NS
Temporal association	101 (34.6)	380 (36.4)	0.87	NS
Sequela of CVD	128 (43.8)	469 (44.9)	0.91	NS
**Vascular risk factors**				
Hypertension	171 (58.6)	493 (47.2)	1.64	<0.001
Diabetes	111 (38.0)	289 (27.7)	1.63	0.001
Hyperlipidemia	97 (33.2)	230 (22.0)	1.83	<0.001
Heart diseases	54 (18.5)	104 (9.9)	2.10	<0.001
**Current medication**				
Anti-dementia drugs	21 (7.2)	63 (6.0)	1.14	NS
Anti-platelets	113 (38.7)	379 (36.3)	1.11	NS
Anti-Parkinson drugs	48 (16.4)	15 (1.4)	13.34	<0.001
Antipsychotics	20 (6.8)	30 (2.9)	2.48	0.002

*CVD, cerebrovascular disease; CDR, Clinical Dementia Rating scale; n, number of cases; NS, Non-significance; OR, odds ratio; Heart diseases, including coronary artery disease, valvular heart disease, congestive heart failure, and arrhythmia.*

Comparison of neuroimaging manifestations between the tremor group and the non-tremor group after adjustment for age and dementia severity according to CDR is demonstrated in Table 4. None of the MRI variables including Fazekas scale, global atrophy scale, medial temporal lobe atrophy scale, or microbleeds showed significant difference between the groups. The comparison of TRODAT variables also showed no significant difference in striatal background ratio, caudate putamen ratio, or rates of abnormal uptake between the groups.

**TABLE 4:** Neuroimaging manifestation of tremor group compared to non-tremor group adjusted for age and dementia severity by CDR.

	Tremor	Non-tremor	OR	*p*
MRI, *n*	194	669		
Fazekas, mean (SD, range)	2.0 (1.0, 0–3)	1.8 (1.0, 0–3)	1.29	NS
MTA, mean (SD, range)	2.3 (1.1, 0–4)	2.2 (1.2, 0–4)	0.96	NS
Global, mean (SD, range)	1.6 (0.7, 0–3)	1.5 (0.8, 0–3)	0.73	NS
Cortical CBM, *n* (%)	47 (23.4)	313 (27.8)	0.70	NS
Subcortical CBM, *n* (%)	65 (32.3)	230 (33.2)	0.97	NS
Trodat, *n*	70	63		
DaTabN, *n* (%)	31 (44.3)	37 (58.7)	0.63	NS
SBR, mean (SD, range)	1.5 (0.5, 0.2–2.8)	1.5 (0.4, 0.5–2.4)	1.29	NS

*CDR, Clinical Dementia Rating scale; n, number of cases; OR, odds ratio; NS, non-significance; Fazekas, Fazekas scale; MTA, medial temopral lobe atrophy scale; Global, global atrophy scale; CMB, cerebral microbleeds; Trodat, Tc-99m Trodat-1 SPECT; DaTabN, abnormal dopamine transporter imaging in Tc-99m Trodat-1 SPECT; SBR, Striatal background ratio in Tc-99m Trodat-1 SPECT; CPR, caudate putamen ratio in Tc-99m Trodat-1 SPECT.*

We further compared clinical features between resting tremor group (*n* = 193) and isolated postural/action tremor group (*n* = 99). The results showed that no significant different between two groups in most of the motor and non-motor features except for bradykinesia (67.9 vs. 51.5%, OR = 2.01, *p* = 0.006) after adjustment for age and CDR. Motor features were also compared according to tremor onset asymmetry or not and the asymmetric group demonstrated significantly higher prevalence of bradykinesia (70.7 vs. 56.8%, OR = 1.87, *p* = 0.014), rigidity (62.1 vs. 47.7%, OR = 1.90, *p* = 0.009), postural instability (81.0 vs. 67.6%, OR = 2.08, *p* = 0.011), and easily falling (28.4 vs. 14.8%, OR = 2.39, *p* = 0.004). Besides, we also analyzed the clinical features among patients with tremor. Patients were divided into groups according to the temporal sequence of tremor and CVD. The results showed that the group showing tremor onset earlier than CVD onset had significantly higher prevalence of REM sleep behavior disorder than the group with later or concomitant onset (23.8 vs. 10.6%, OR = 2.66, *p* = 0.004). No other features were significantly related to the temporal sequence of tremor onset and CVD.

## Discussion

The present study has several important findings. A relatively high prevalence of resting and/or postural/action tremors (21.8%) was found in patients with VCI. This finding was not consistent with the findings of previous studies, which reported that the association of movement disorders and stroke was uncommon and only 1% of the patients developed movement disorders after acute stroke (Ghika-Schmid et al., 1997). This discrepancy in the results was probably due to the fact that motor deficit due to acute stroke tends to improve after the development of abnormal movement (Handley et al., 2009). However, our study investigated CVD patients with cognitive impairment without restricting the investigation to acute CVD. Therefore, the present study population was different from that in the previous studies. Moreover, tremors might have started before CVD and persisted without improving. The comparison of VCI patients in the tremor group and those in the non-tremor group showed no difference in the subtype of VCI (MCI or dementia stages) or in the location of CVD (cortical, subcortical, mixed location, infarct, or hemorrhage). These findings indicated that tremor in VCI patients was probably not simply caused by vascular disease itself. Instead, degenerative process might have contributed the most to the pathophysiology or the etiology of tremor. Furthermore, rather than atypical tremors related to acute stroke (Ghika-Schmid et al., 1997; Handley et al., 2009), VCI patients presented high prevalence of typical (resting and postural/action) tremors, which are traditionally regarded as tremors of neurodegenerative disorders (Zappia et al., 2013; Postuma et al., 2015; McKeith et al., 2017). Therefore, mixed vascular and degenerative pathologies were also implicated in this group of VCI patients with typical tremor. The longer duration of motor dysfunction in the tremor group compared to the non-tremor group represented a higher percentage of early onset of motor dysfunction (before the onset of CVD or VCI). In other words, among patients with tremor, movement disorders were diagnosed before the diagnosis of CVD or VCI. Besides, the much higher prevalence of motor and non-motor diagnostic features of LBD in the tremor group provided further evidence of the possibility of the prevalence of mixed pathologies with LBD being higher in the tremor group. High percentage of abnormal dopamine transporter imaging studies and no difference in striatal uptake is reported between patients with and without tremor. We proposed that structure damage in the striatal region might probably contribute to the impairment of uptake of the dopamine transporter imaging. Besides, patients with clinical pictures of mixed Parkinson’s disease were prone to have a dopamine transporter imaging. Therefore, abnormal uptake in both groups were relatively high in this study (44.3% in tremor and 58.7% in non-tremor group).

The higher prevalence of vascular risk factors (VRFs) including hypertension, diabetes, hyperlipidemia, and heart diseases in the tremor group were interesting and novel findings in VCI patients. Previous studies on the association between VRFs and VCI revealed that these factors contributed to the cardio-vascular diseases (CVD) attack or to the occurrence of cognitive impairment/dementia (Jeng et al., 1994; MacKnight et al., 2002; Schulz and Rothwell, 2003; Gorelick, 2004; Sahathevan et al., 2012; Staals et al., 2014). Besides, most of the midlife VRFs have been associated with increased neurodegenerative or vascular disorders and cognitive impairment as a comorbidity in late life (Kivipelto et al., 2001; Whitmer et al., 2005; Barnes et al., 2012). The contribution of VRFs has been well studied in patients with movement disorders. Patients with PD are associated with lower prevalence of VRFs or CVD (Scigliano et al., 2006). CVD and VRFs show significant association with motor and cognitive dysfunction in PD (Papapetropoulos et al., 2004; Malek et al., 2016). Therefore, these research findings might help explain our results of higher prevalence of VRFs in VCI patients with tremor. Furthermore, we proposed that VRFs contributed not only to the incidence of CVD and cognitive impairment but also to the incidence or progression of tremor-related movement disorders like LBD. For better clarification, we further analyzed the motor composite questionnaire (MCQ) (total score 6, Table 2) as well as the non-motor composite questionnaire (NMCQ) (total score 10, Table 2) in the present study and found that in the current VCI population, MCQ and NMCQ were higher in the hypertension group compared to the non-hypertension group (MCQ: 2.0 ± 1.8 vs. 1.5 ± 1.7, *p* < 0.001 and NMCQ: 2.8 ± 2.4 vs. 2.2 ± 2.3, *p* < 0.001); higher in the diabetes group than in the non-diabetes group (MCQ: 2.1 ± 1.8 vs. 1.6 ± 1.8, *p* < 0.001 and NMCQ: 3.0 ± 2.4 vs. 2.3 ± 2.4, *p* < 0.001); higher in the hyperlipidemia group than in the non-hyperlipidemia group (MCQ: 2.1 ± 1.8 vs. 1.6 ± 1.8, *p* < 0.001 and NMCQ: 2.9 ± 2.3 vs. 2.3 ± 2.4, *p* < 0.001); and higher in the heart diseases group than in the group with no heart diseases (MCQ: 2.3 ± 1.8 vs. 1.7 ± 1.8, *p* < 0.001 and NMCQ: 3.3 ± 2.4 vs. 2.4 ± 2.4, *p* < 0.001). In addition to age and dementia severity, we further adjusted gender, antiplatelet, and Fazekas scale, and the results were still the same as our original ones. Based on these findings, we may conclude that each VRFs could have contributed to the presentation of motor as well as non-motor Lewy body features in patients with VCI.

Finally, it deserves attention that in this study, more than 80% of patients in the tremor group present parkinsonism, but only 16.4% were treated with antiparkinsonian drugs, indicating that neurologists mostly consider this neurological condition as a vascular parkinsonism, probably preventing patients from having a possibly effective treatment. Careful and detailed clinical assessment of associated symptoms in VCI patients with tremor to find out the possible comorbidity with PD or DLB should always be in mind.

The present study has some limitations. It was a retrospective study without pathological confirmation of the diagnosis. Therefore, the findings and the speculations were based only on clinical observations and neuroimaging. Clinical presentations in the present study were recorded based on a structured questionnaire, and though the interrater reliability test showed good reliability, accuracy and completeness of the clinical data needs to be verified. Only 10% of the participants in the study had received dopamine transporter imaging. Therefore, the reported contribution of LBD to VCI was based only on the clinical features. Further prospective studies based on biomarkers for LBD (CSF/plasma α-synuclein and/or dopamine transporter imaging) are necessary to clarify the probability and the contribution of Lewy body pathology to VCI with tremor.

In conclusion, tremor is a common comorbidity of VCI. VCI patients with tremor had a higher prevalence of all motor and non-motor Lewy body clinical features and more severe neuropsychiatric symptoms. These findings raised the possibility of mixed pathology with LBD in VCI patients with tremor. Importantly, VCI patients with tremor showed higher prevalence of VRFs including hypertension, diabetes, hyperlipidemia, and heart diseases. However, causal relationship of VRFs in VCI with parkinsonism needs further study to be clarified.

## Data Availability Statement

The data analyzed in this study was subject to the following licenses/restrictions: The raw data supporting the conclusions of this article will be made available by the authors, without undue reservation, to any qualified researcher. Requests to access these datasets should be directed to P-YC, paiyibox@gmail.com.

## Ethics Statement

The studies involving human participants were reviewed and approved by Institutional Review Board of Show Chwan Memorial Hospital. Written informed consent for participation was not required for this study in accordance with the national legislation and the institutional requirements.

## Author Contributions

P-YC undertook the literature search and data analysis, edited the author contributions, and was mainly responsible for revisions and drafts of the manuscript. C-JH participated in the data analysis and contributed to revisions and the final draft of the manuscript. C-YW participated in data analysis and contributed to revisions of the manuscript. G-UH contributed to revisions of the manuscript. R-CT undertook the literature search and contributed to revisions. All authors contributed to the article and approved the submitted version.

## Conflict of Interest

The authors declare that the research was conducted in the absence of any commercial or financial relationships that could be construed as a potential conflict of interest.
